# Comparison of Cytokine Expression Profile in Chikungunya and Dengue Co-Infected and Mono-Infected Patients’ Samples

**DOI:** 10.3390/pathogens10020166

**Published:** 2021-02-04

**Authors:** Saravana Murali Krishnan, Jayashri Mahalingam, Shanthi Sabarimurugan, Thiruvengadam Muthu, Baskar Venkidasamy, Kaveri Krishnasamy, Ashutosh Sharma, Sathishkumar Ramalingam

**Affiliations:** 1 Plant Genetic Engineering Laboratory, Department of Biotechnology, Bharathiar University, Coimbatore 641046, India; saravanamuralik@gmail.com (S.M.K.); baskarbt83@gmail.com (B.V.); 2 Department of Virology, King Institute of Preventive Medicine and Research, Chennai 600032, India; kaveri1971@gmail.com; 3 Zoonosis Research Center, Department of Infection Biology, School of Medicine, Wonkwang University, Iksan 570749, Korea; 4 Institute of Health Education and Translational Sciences, Hyderabad 500026, India; mjayashri@gmail.com; 5 VRR Institute of Biomedical Sciences, Madras University, Chennai 600116, India; 6 School of Biomedical Sciences, University of Western Australia, Crawley, WA 6009, Australia; Shanthi.sabarimurugan@uwa.edu.au; 7 Department of Applied Bioscience, Konkuk University, Seoul 05029, Korea; muthu@konkuk.ac.kr; 8 Centre of Bioengineering, Technologico de Monterrey, Epigmenio Gonzalez #500, Fracc. San Pablo, Campus Queretaro, Santiago de Querétaro 76130, Queretaro, Mexico

**Keywords:** Chikungunya, Dengue viral load, inflammatory cytokines, ELISA, RT- PCR

## Abstract

Chikungunya (CHIKV) and Dengue (DENV) viruses cause an acute febrile illness which is hard to clinically differentiate and treat since both exhibit similar symptoms. Hence, this study was aimed at identifying the expression profiles of cytokines on co-infected samples and compare with CHIKV and DENV mono-infected samples. Serum samples of 292 suspected patients during 2009–2011 were analyzed. The presence of primary (IgM)/secondary (IgG) antibodies and early NS1 Dengue antigens were confirmed by capture ELISA. Molecular diagnosis and serotypes were discriminated by RT-PCR, confirmed by sequencing. All the plasma samples were assayed for cytokine expression by BDTM cytometry bead array (CBA) and compared with independent mono-infection viral load. Among the tested samples, 82 were confirmed as Dengue positive; 52 through IgM (17.8%), and 30 through IgG (10.2%). Additionally, 186 samples were confirmed as Chikungunya, 96 through IgM (32.6%) and 92 through IgG (31.5%) ELISA, respectively. Interestingly, 19 patients were co-infection positive in which, only 6 were confirmed for CHIKV and 7 for DENV by RT-PCR. Among 8 cytokines, IL-2, IL-8, IFNα, IFN γ, and IL-12 were found to be significantly different between co-infected and CHIKV mono-infected patients and correlated with viral load. DENV viral load was correlated with cytokine expression and a significant difference in IL-2 and IL-12 was observed between DENV mono-infected and DENV and CHIKV co-infected patients. Results indicated that apart from serological and molecular confirmation, cytokines could be used as a specific biomarker for the diagnosis of DENV and CHIKV. In the future, the role of independent cytokines can be determined to understand the pathogenesis and etiology of these dreadful diseases.

## 1. Introduction

Dengue and Chikungunya are neglected tropical arboviral diseases that are transmitted by *Aedes aegypti* and *Aedes albopictus* mosquitoes. Both the viruses often co-circulate through the common vector and are transmitted to humans as co-infections [1]. The occurrence of Dengue (DENV) and Chikungunya (CHIKV) epidemics are found to occur in higher magnitude during cold, rainy, and humid weather conditions due to relatively high propagation of the mosquitoes [2]. According to WHO, the prevalence of Dengue has been on the rise in South East Asia since 2007 with an increase of 18% in Dengue cases [3,4,5]. Since 2005, there has been a resurgence of CHIKV, especially in Southern India. Incidence has recently increased in the USA, Southeast Asia, Europe, and the Pacific Islands as well [6]. Ironically, a recent report shows CHIKV infection even during dry summer months [7]. Even though DENV cases are documented yearly, the total burden is unclear which necessitates increased vector surveillance. The clinical presentations of DENV and CHIKV are found to be very similar. Both these infections are not easy to differentiate due to the overlap of many clinical signs and symptoms [8,9]. Anecdotal reports show that changing platelet count serves as a laboratory parameter of DENV [10] with the availability of specific tests for serotype identification through RT-PCR or other preliminary confirmation done by ELISA. Molecular serotyping is the only option to differentiate the variants due to their difference in heterogeneity. Due to the unavailability of antiviral drugs or vaccines, there is a dire need to develop better disease-specific diagnostic biomarkers for DENV and CHIKV infections and it will help in case management. To date, there is no comprehensive study related to the two clinically similar conditions [11]. Since both DENV and CHIKV have similar clinical presentations, it is worthwhile to determine the biomarkers associated with each of the specific diseases [12,13]. DENV and CHIKV viruses are likely to coexist in the vector before infection and coexist in the same host, causing a varying degree of clinical manifestations in the patient [14,15]. DENV and CHIKV co-infections were reported in 1967 in Calcutta, India. To date, ELISA is the preliminary diagnostic method and co-infection confirmed by RT-PCR [16]. In ELISA, the monoclonal antibody is used to differentiate the DENV and CHIKV infection in diagnostic methods. In RT-PCR, viral load and genotyping are used to establish the property of the virus and heterogeneity [17]. During the acute phase of CHIKV and DENV infection, the correlation of virological and immunological markers to cytokine levels is likely to provide insight into the mechanism of immune response and pathogenesis [18]. However, an effective predictive biomarker is still needed to assess the progression and severity of the disease. Therefore, in the present study, the evaluation of cytokine expression profiles in acute stages of the DENV and CHIKV infections may contribute towards identifying disease-specific diagnostic biomarkers for both the viral diseases. These biomarkers may help in not only identifying DENV and CHIKV infection but also help to distinguish the disease progression and severity and case management. 

## 2. Results

### 2.1. Study Population and Demographic Details

Patients were screened for the clinical manifestations of: fever, 292 (100%); myalgia, 243 (83.2%); vomiting, 95 (35.2%); headache, 256 (87.6%); arthralgia,266 (84.9%); hemorrhagic manifestations including petechiae, 212 (72.6%); diarrhea, 25 (8.5%); hematuria, 12 (4.1%); and skin rashes, 14 (4.7%) (Table 1). The most common symptoms of CHIKV are swelling of the joints and crippling joint pain affecting fingers, knee, shoulder, ankles, and toes. A febrile phase, consisting of fever, maculopapular rash, arthralgia, myalgia, and thrombocytopenia, is common in DENV infection. Males (130) and females (162) between age 22 and 65 years along with 60 age-matched healthy controls were included in the study. Males (30) and females (30) between the ages of 22 and 65 years were included as healthy control.

### 2.2. Viral Load and Serological Findings

In the 292 samples, 286 were subjected to CHIKV and DENV IgM ELISA. Four samples were inadequate and two were lysed and could not be used for laboratory diagnosis methods. IgM and IgG ELISA and RT-PCR were used as laboratory diagnostic methods to identify CHIKV and DENV mono and co-infections. In addition, NS1 ELISA was also used to identify early infection of DENV. Among the 286 patients, 96 (33.5%) were IgM positive for CHIKV, 41 (14.3%) were DENV IgM positive, 9 (3.1%) were positive by NS1 and IgM ELISA, 2 (0.7%) were positive by NS1 ELISA and RT-PCR, 92 (32.2%) samples were IgG positive for CHIKV, 30 (10.5%) were DENV IgG positive, and 16 (5.5%) samples were identified as IgM positive in both DENV and CHIKV. By RT-PCR, 6 (2.05%) were positive for DENV, 7 (2.4%) were positive for CHIKV, and 6 (2.05%) were positive for both. The cytokine profiles were analyzed and correlated with viral RNA of DENV and CHIKV in the samples collected between days 2 and 14 of the illness.

### 2.3. CHIKV, DENV Mono-Infections and CHIKV and DENV Co-infections Are Associated with an Inflammatory Cytokine Profile

The inflammatory cytokine milieu was described in DENV, CHIKV, and DENV and CHIKV co-infection (Table 2). Pro-and anti-inflammatory cytokines in the acute phase of DENV, CHIKV infection, and DENV and CHIKV co-infection were profiled. Blood samples were collected from 185 CHIKV, 82 DENV, and 19 DENV and CHIKV co-infected patients. All the 19 co-infected samples were based on IgM ELISA and RT-PCR. Thirty-five plasma samples were separated from the blood and the mean level of cytokines was determined by ELISA. All the 35 samples were negative for IgG ELISA. Of the 8 cytokines, IL-2, IL-6, IL-10, IL-12, and IFNα (*p* = 0.0002 for IL-2; *p* = 0.007 for IL-6; *p* = 0.0001 for IL-12; *p* = 0.02 for IL-10; and *p* = 0.0003 for IFNα) were significantly elevated in DENV patients as compared to healthy controls. IFNγ was slightly elevated, but IL-8 and TNFα did not show a significant increase in DENV patients. Among the 8 cytokines, the levels of 5 cytokines (*p* < 0.0001 for IL-6; *p* < 0.0001 for IL-10; *p* = 0.004 for IFNα; *p* = 0.002 for TNFα; and *p* = 0.0002 for IFN-γ) were significantly elevated in CHIKV patients as compared to the healthy controls. However, IL-2 and IL-12 did not show a consistent increase as compared to the controls. Cytokine profile IL-6, IL-10, IL-12 and IFN-γ (*p* = 0.04 for IL-6; *p* = 0.02 for IL-10; *p* = 0.03 for IL-12; and *p* = 0.02 for IFN-γ) were significantly increased in the CHIKV and DENV co-infected group as compared to the control group.

### 2.4. Correlation between DENV and CHIKV Viral Load and Inflammatory Cytokines

**Figure 1:**
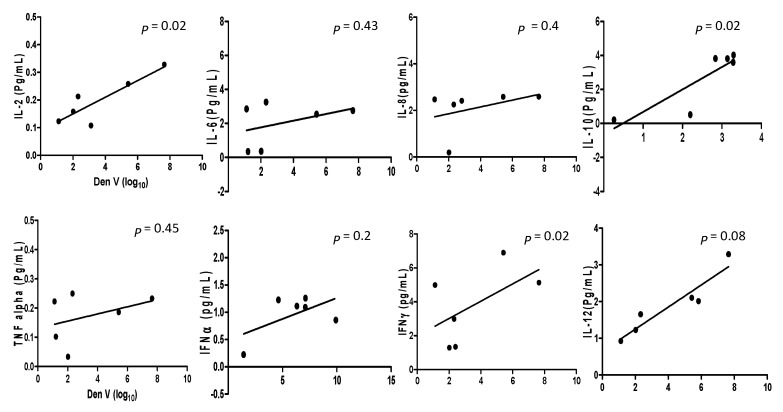
The cytokine expression levels in Dengue mono-infection and co-infection samples are correlated with regression analysis and the scatter points represent the variable between the sample’s cytokine expression level and viral copy number of Dengue infection. The results are expressed from three independent experiments.

**Figure 2:**
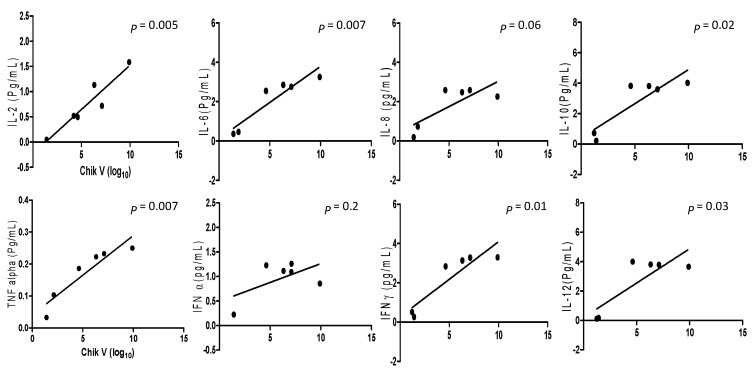
The cytokine expression level between Chikungunya mono-infection and co-infection samples correlated with regression analysis and the scatter points represent the variable between the sample’s cytokine expression level and viral copy number of Chikungunya infection. The results are expressed from 3 independent experiments.

A comparison was drawn between viral loads and inflammatory cytokines levels in DENV and CHIKV patients. Analysis of pro-and anti-inflammatory cytokine levels with the DENV positive samples collected within the acute phase of infections revealed that IL-2 and IL-10 (*p* = 0.02, r2 = 0.759 for IL-2 and *p* = 0.02, r2 = 0.748 for IL-10) had significant correlation with the viral RNA copies (Figure 1). When comparison was made between CHIKV positive samples and their inflammatory cytokine expression levels, it was observed that IL-2, IL-6, IL-10, TNF-α, IFNγ, and IL-12 (*p* = 0.005, r2 = 0.88; *p* = 0.007, r2 = 0.85; *p* = 0.02, r2= 0.76; *p* = 0.0077, r2 = 0.86; *p* = 0.01, r2 = 0.81; and *p* = 0.03, r2 = 0.69 respectively) were significantly correlated with CHIKV viral load (Figure 2).

### 2.5. Comparison of Cytokine Level between DENV and CHIKV Co-Infection, DENV and CHIKV Mono-Infection

**Figure 3:**
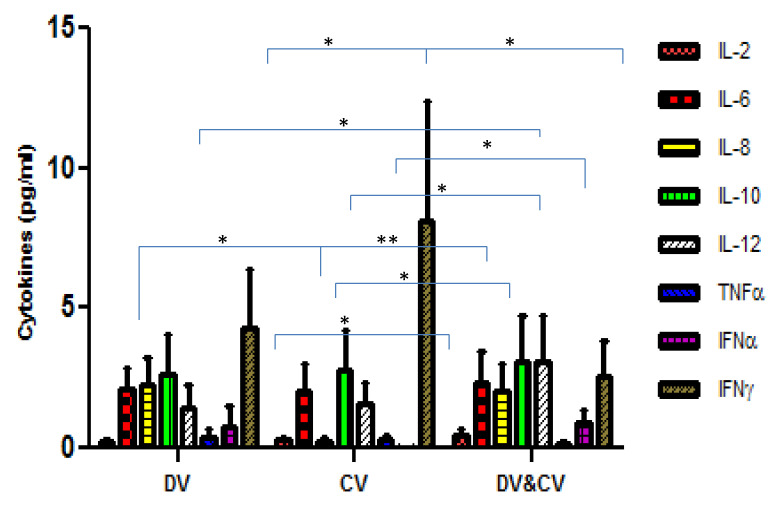
The bar diagram depicting the level of cytokines amongst the Dengue virus-infected patients (DV), Chikungunya infected patients (CV), and Dengue and Chikungunya co-infected patients. The X-axis represents the DV, CV, and DV and CV co-infected patients. Y-axis represents the level of cytokines in Pg/mL. *p*-value < 0.05 (*); *p*-value < 0.005 (**) were considered significant.

The comparison of cytokine levels in the plasma samples of DENV and CHIKV mono-infected and DENV and CHIKV co-infected patients are shown in Figure 3. Of the 8 cytokines, a significant difference was found for IL-2, IL-8, IFNα, IFNγ and IL-12 (*p* = 0.01; *p* = 0.01; *p* = 0.003; *p* = 0.01; *p* = 0.02 respectively) between CHIKV mono-infected and DENV and CHIKV co-infected patients. Significant differences were observed for IL-8 and IFNα (*p* = 0.004; 0.02, respectively) between DENV and CHIKV mono-infected patients. IL-2 and IL-12 (*p* = 0.03; 0.03 respectively) levels were found to significantly vary between DENV mono and co-infected patients.

## 3. Discussion

Chikungunya and dengue are serious life-threatening arboviral infections causing morbidity and mortality in India especially in the tropical regions of South India. In a previous study, Chahar et al. [19] identified that 17 out of 69 serum samples were positive for CHIKV by RT-PCR; 6 samples positive for both viruses and suggested that they are co-spreading and can be transmitted together [19]. Comparably, our results show that 19 DENV/CHIKV co-infected samples were identified as IgM Positive and 6 were confirmed for DENV and CHIKV infection by RT-PCR. The virus can be detected from serum, plasma, blood cells, and tissues based on the onset of illness [20] A few reports revealed the episodes of occurrence of co-infection in India [21,22,23,24]. It was shown that two infections could coexist in the same host, *A. aegypti* and *A. albopictus* transferring the two viral infections along with identical clinical symptoms [25,26]. The Chantal et al. [27] study showed that an increase in the possibility of co-transmission from *A. aegypti* to humans makes an important contribution to the burden of co-infection during overlapping outbreaks. Since there is a serious threat of both the infections spreading in tropical regions, better supervision to diagnose and differentiate these infections and molecular level examination are needed for proper management. 

In the present study, the immune responses of acute phases of CHIKV and DENV patients were examined and compared to find out disease-specific diagnostic biomarkers for both diseases. In our analysis, increasing and declining tendencies of varying cytokines in DENV and CHIKV infections were observed. In the acute stage of the DENV infection, the levels of the cytokines, such as IL-2, IL-6, IL-8, IL-10, IL-12, and IFNα were increased whereas, in the acute phase of CHIKV infection, cytokines IL-6, IL-10, TNFα, IFNα, and IFNγ showed increasing tendencies. Varied cytokine expression profiles of IL-10 and IFNγ in DENV infected patients were observed. IL-10 shows a prominent role in the immune response by suppressing the viral disease via activation of NK cells and by triggering immunoregulatory and anti-inflammatory cytokines against the antigen by associating with mast cells, B cells, and certain T cells. Besides, IFNα is derived from leucocytes, targeting various active host immune cells including macrophages which inhibit viral replication [22].

Our results are in accordance with an earlier report of Rojas [28]. It was concluded that IL-2, IL-12, IFNα, and IL-6 increased in the acute stage of DENV. Further, IL-6 is a significant immune mediator for fever and is known to stimulate muscle metabolism to enhance body temperature during the acute phase of CHIKV infection. This effect is comparable to that of rheumatoid arthritis, causing joint inflammation and pain [29,30]. A previous study revealed that decreasing trends of IL-2, IL-12, and TNFα were observed in the acute phase of CHIKV infection and increased during recovery [31]. Similarly, TNFα, IL-10, IL-12, and IFN-γ increased in the convalescent phase of CHIKV infection [32]. A few reports indicated that depending upon the cytokine elevation, the immune response may get activated biologically [31,33]. Reports suggest that every cytokine plays a significant role in the immune response in the acute and convalescent-phases of infection [31,32,33,34]. In the present study, results revealed exclusive cytokine expression among the freshly isolated samples and also analyzed the differential expression in mono-infection and co-infection samples. Our findings strongly suggest a robust cytokine response during acute infections. 

Clinical pictures of the acute phase of DENV and CHIKV mono-infections were framed and correlated with the viral load which represented that the inflammatory cytokine profile is playing a pivotal role in clinical features of DENV and CHIKV mono and co-infections. Many previous studies showed that IL-6 and IL-10 were found as a marker for disease severity in DENV and CHIKV infections [35]. Our results indicated that increased inflammatory cytokine profiles correlated with the viral load in the acute phase of DENV or CHIKV mono-infection. Additionally, in the co-infection, a higher viral load is found and correlated to higher cytokine levels. However, the Jaspreet Jain [36] study revealed that the expression of high viral load can be used as a surrogate marker for identifying the post-acute phase/chronic phase of infection and disease severity. The ROC curve was also analyzed to calculate the potential value of these cytokines in mono and co-infections (Supplementary Figure S1). The AUC of cytokines was greater than 0.7188 which indicated a high diagnostic value. This regression model included inflammatory cytokine profiles (IL-2, IL6, TNFα, and IFN α) with a strong predictable probability and disease severity. Accordingly, it is inferred that these cytokines might play a pivotal role in inflammatory diseases, and it could be considered as a significant biomarker candidate for immunotherapeutic measures. Further, a large cohort of patients was included in the present study for evaluating the validity of the diagnostic parameters for mono and co-infections, and the results related to the occurrence of the epidemic were analyzed comparatively. The evaluated levels of cytokine expression were compared with the viral load of individual samples. These results contribute to an improvised analysis to know the diagnostic accuracy of cytokine expression levels from DENV and CHIKV infected patients. Further studies on these lines would lead to evolving a specific diagnostic biomarker for these infections. There is a limitation in this study—samples were collected at only one point in time. If the profiles of cytokines and chemokines are analyzed during the acute, chronic, and recovery phase of infections, it may provide better insight into the immune modulations mediated by them. Further, a detailed study about the predictive diagnostic markers might help in the direction of management of severe cases and anticipation of complications.

## 4. Materials and Methods

### 4.1. Clinical Specimens

During the 2009–2011 outbreaks, 292 clinically suspected samples were collected from the Government Hospital, Tirunelveli district, Tamil Nadu. The patients with clinical manifestations, such as arthralgia, myalgia, headache, fever, thrombocytopenia, skin rashes, and hemorrhagic fever were selected and subsequently diagnosed for DENV and CHIKV. Clinical signs and symptoms were also assessed in the selected patients (WHO criteria, 2009). The onset of illness was recorded as per the disclosure by the patients. Blood samples were collected at the acute phase of the disease starting from day 2 to 14 after the onset of illness. The initial 2 to 5 days and chronic phases of both the infections were registered and analyzed by NS1 Ag detection, RT-PCR, and cytokine analysis. Chronic samples were chosen for ELISA IgM and IgG diagnostics. The aseptically collected blood samples were sent to the King Institute of Preventive Medicine and Research (State Virology Laboratory, KIPM and R). After the separation of serum and plasma samples, preliminary and confirmatory diagnostic protocols for both the diseases were carried out. The serum samples were subjected to ELISA and RT-PCR and the plasma samples for cytokine expression. Thirty-five samples that were collected in the acute phase were subjected to cytokine analysis. Among the 35 samples, 19 were co-infected. 

### 4.2. Sample Processing 

The whole blood samples were segregated for serological investigation and the plasma samples were employed for molecular analysis. Aliquots of the serum and plasma samples were taken in sterile vials and stored at −80 °C. Ten samples from healthy individuals (with no signs and symptoms) were used as a negative control. All samples were handled and processed according to Institutional Biosafety Guidelines, Biosafety level 2 (BSL-2).

### 4.3. Enzyme-Linked Immunosorbent Assay 

The IgM and IgG kits were procured from the National Institute of Virology, Pune and the NS1 ELISA Kit was procured from Panbio (Inverness Medical Innovation, Australia). The diagnostic assays were performed according to the manufacturer’s instructions.

#### 4.3.1. IgM ELISA for CHIKV and DENGUE

The IgM ELISA procedure was performed as per the manufacturer’s protocol [37]. The IgM ELISA for CHIKV was processed as follows: sample dilution buffer (495 µL) and sample (5 µL) was added in 96 well plates and vortexed for 5 s. To 50 µL of the diluted samples, controls were added and incubated at 37 °C for 1h in the dark. The contents were then discarded and washed with washing buffer and 50 µL of CHIKV antigen was incubated for 1 h at 37 °C in the dark. After washing, 50 µL of monoclonal antibodies were added and incubated at 37 °C. 50 µL of HRP conjugate was added after washing and incubated for 30 min at 37 °C. Substrate (100 µL) was added after washing and incubated for 10 min at 37 °C. Finally, 100 µL of stop solution (H_2_SO_4_) was added and plates were read under ELISA reader at 450 nm. The IgM ELISA for Dengue was performed according to Shanthi et al. [38].

#### 4.3.2. IgG ELISA for CHIKV and DENGUE

Microtitre wells (Nunc) coated with human anti-CHIKV and anti-DENV antigen were placed at room temperature (RT) and 100 μL of standards, controls, and the samples (CHIKV and Dengue) were added and incubated at RT for 1 h. Peroxidase-labeled anti-Dengue/anti-CHIKV monoclonal antibodies (125 μL) were added and incubated for 1 h at RT. The contents were then aspirated and blot dried after adding secondary antibodies. Finally, a substrate solution (100 μL) was added and incubated for 20 min followed by the addition of 100 μL 1N HCL to stop the reaction. The absorbance was measured using an ELISA reader at 450 nm. 

#### 4.3.3. NS1 Antigen Detection for DENGUE Virus

To detect the NS1 antigen with respective controls, the patient serum sample was diluted 1:10 and added to micro-titers wells pre-coated with monoclonal antibodies of NS1 and incubated at 37 °C for 1h. The processed wells were then washed with wash buffer 5 times to remove unwanted serum particles. About 100 μL of anti-NS1 MAb-HRP and conjugate was added and incubated at 37 °C and washed with dilution buffer following which TMB/H_2_O_2_ was added. The plate was incubated for 10 min at RT and the reaction was stopped by the addition of H_2_S0_4_. The absorbance was read at 450 nm.

### 4.4. RT-PCR for Chikungunya Virus by Employing a Sensitivity Improved Method

All samples were tested for Chikungunya virus-specific RNA by RT-PCR. RNA was extracted using the QIA amp Viral RNA Mini Kit (Qiagen, Hilden, Germany). A one-step RT- PCR was performed using Superscript III one-step RT-PCR platinum–Taq kit (Invitrogen, Carlsbad, California), by following the manufacturer’s instructions and with the primer pairs: E2F: TATCCTGACCACCCAACGCTCC (9403bp-9424bp), E2R: CACATCCCACCTGCC (9693bp-9712 bp) targeting the E2 gene designed from the nucleotide sequence of the reference S27 strain [39]. The reaction was carried out in 25 µL containing 12.5 µL 2× reaction mix, 10 µL water, 0.2 µM primers, 0.5 µL RT/Taq, and 1 µL RNA. The PCR conditions were as follows: 50 °C for 30 min, 94 °C for 2 min, 40 cycles of 94 °C 15 s, 55 °C for 30 s, 68 °C for 2.20 min, and 68 °C for 5 min. Real-time PCR was carried out for RT-PCR positive samples to validate and detect a viral load. 

### 4.5. RT-PCR for Dengue Virus

All the samples were subjected to Pan Dengue RT-PCR. The reaction mixture contained 5 μL RNA, 25 pmol of the D1 (119 bp), D2 (288 bp), D3 (260 bp), and D4 (208 bp) primers and components of one-step RT-PCR kit (QIAGEN, Hilden, Germany). The PCR conditions were as follows: 50 °C for 30 min; 95 °C for 15 min, 55 °C for 15 s, and 72 °C for 30 s; 34 cycles at 95 °C for 15 s, 55 °C for 15 s, and 72 °C for 30 s; and 72 °C for 10 min. After amplification, the product (5 μL) was analyzed by agarose gel electrophoresis.

### 4.6. Estimation of Viral Load 

RT-PCR was carried out in a light cycler 2.0 (Roche Diagnostics, Burgess Hill, UK). Ten-fold serial dilution of positive control with an unknown test value was used for the construction of the standard curve for viral load quantification. The CHIKV and DENV final concentration of internal control were observed as approximately 100 to 100,000 DNA copies per mL, equivalent to a threshold cycle (Ct) value of 32 to 38≈. The internal control of the Chikungunya virus final concentration was 100 to 100,000≈ RNA copies per mL, which is equivalent to the Ct value of 30≈ and for Dengue 35 in a real-time detection system [40,41]

### 4.7. Genotyping

The RT-PCR positive amplicons were subjected to double-stranded sequencing, using a big dye terminator cycle sequencing-ready reaction kit on an ABI 310 sequencer (Applied Biosystems, Foster City, CA, USA). The molecular relationship of the partial genome of CHIK and members of the alphavirus for analysis were taken into consideration [8]. The complete genotype of the CHIKV was determined by nucleotide sequencing and compared with the globally diverse CHIK isolates. The specific DNA amplicons were purified (Microcon, Millipore, Madrid, Spain) and sequencing reactions were carried out with the Taq Dye Deoxy Terminator Cycle Sequencing kit (Applied Biosystems, Foster City, CA, USA) and purified using CentriSep columns (Princeton Separations, Adelphia, New Jercy USA). The sequences were resolved with an ABI PRISM 310 Genetic Analyzer (Applied Biosystems, Foster City, California, USA) and processed using the DNASIS 3.6 Software, Mac version (Hitachi, Japan).

### 4.8. Immunoassay for Cytokine Expression 

The respective CHIKV and DENV infected samples and the co-infected samples were analyzed for cytokine levels with 10 healthy controls using BDTM Cytometry Bead Array (CBA) Human Cytokine kit for the following biomarkers: IL-2, IL-6, IL-8, IL-10, IFNα, IFNγ, and IL-12. Briefly, the individual standard curve range for a given cytokine defines the minimum and maximum quantifiable level using the BD CBA Human Th1/Th2 Cytokine Kit (BD Bioscience) and the protocol was adopted according to the manufacturer’s instruction. Plasma samples were incubated for 3 h after adding PE detection reagent with capture beads and washed with wash buffer and analyzed on a flow cytometer [42].

### 4.9. Statistical Analysis

All in vitro experiments were analyzed in triplicates. The IgM, IgG, NS1 detection, and cytokine expression level were analyzed as per the mentioned standardized protocols. The results of experiments were statistically analyzed using SPSS software 17.0 (IBM, India)and the values presented as mean ± SD where *p* < 0.05 was considered statistically significant.

## 5. Conclusions

Dengue and Chikungunya are two major vector-borne diseases and unfortunately, there is neither vaccine nor any appropriate antiviral therapy to treat both infections. Therefore, the diagnosis of the infection at the early stage is found to be necessary for better clinical management of the twin diseases. The results of the present study revealed that IL6, IL10, and IFNα were elevated in DENV and CHIKV mono-infected samples. IFNγ was significantly upregulated in CHIKV and IL-2, IL-8, and IL-12 significantly increased in DENV infection. In the present study, diagnostic accuracy in employing cytokine as a potent diagnostic biomarker for DENV and CHIKV infections was evaluated. Further, the cytokine expression level has been observed from a large cohort of DENV and CHIKV patients and its level of significance has been rigorously evaluated. The IL2, IL10, IFNγ, IFNα, and IL12 were elevated in DENV and CHIKV coinfected samples. IFNα and IL-12 were upregulated significantly in CHIK and DENV co-infections than mono-infection. Hence, further studies on the role of cytokines and their molecular mechanism of action will contribute to a deeper understanding of the twin diseases and the possibility of using cytokines as a predictive diagnostic and prognostic biomarker for DENV and CHIKV viral infections. 

## Figures and Tables

**Table 1:** Symptom characteristics of Chikungunya (CHIKV)- and Dengue (DENV)-infected patients with observed clinical manifestation.

Symptoms	Number of Cases	Percentage
Fever	292	100
Myalgia	243	83.2
Vomiting	95	35.2
Headache	256	87.6
Arthralgia	266	84.9
Hemorrhagic manifestation with petechiae	212	72.6
Diarrhea	25	8.5
Hematuria	12	4.1
Skin rashes	14	4.7

**Table 2:** Comparison of cytokine levels from CHIKV, DENV, and CHIKV and DENV co-infected samples.

	Control Mean ± SD (Pg/mL)	CHIKV Infected Patients Group Mean ± SD	DENV Infected Patients Group Mean ± SD	DENV and CHIKV Co-Infected Patient Group Mean ± SD
IL-2	11.08 ± 10.05	7.2 ± 6.4	16.25 ± 10.19	17.44 ± 13.19
IL-6	12.9 ± 11.4	15.2 ± 94.9	16.4 ± 11.0	18.27 ± 12.42
IL-8	5.3 ± 1.8	5.4 ± 2.8	9.09 ± 1.4	8.88 ± 3.53
IL-10	1.2 ± 0.8	4.7 ± 4.5	3.7 ± 2.5	5.01 ± 3.92
TNF-α	1.2 ± 0.8	2.6 ± 5.9	1.5 ± 0.9	2.18 ± 0.079
IFN-α	0.6 ± 0.2	7.8 ± 9.4	11.6 ± 9.6	12.92 ± 10.38
IFN-γ	3.12 ± 2.1	15.2 ± 4.5	4.4 ± 4.3	3.91 ± 2.32
IL-12	9.7 ± 8.5	8.8 ± 7.4	12.63 ± 10.6	16.05 ± 12.46

## Data Availability

The data presented in this study are available on request from the corresponding author. The data are not publicly available due to privacy.

## Supplementary Materials

The following are available online at https://www.mdpi.com/2076-0817/10/2/166/s1, Figure S1: ROC.

## References

[1] Vikram L., Sachee A., Nilima V., Seema K., Shashtri J.S., Sujatha S. (2016). Dengue and Chikungunya virus co-infections: The inside story. J. Assoc. Physicians India.

[2] Dhara V.R., Schramm P.J., Luber G. (2013). Climate change and infectious diseases in India: Implications for health care providers. Indian J. Med. Res..

[3] Guzman M.G., Kouri G. (2002). Dengue: An update. Lancet Infect. Dis..

[4] Gubler D.J. (2011). Dengue, urbanization and globalization: The unholy trinity of the 21st century. Trop. Med. Health.

[5] Undurraga E.A., Halasa Y.A., Shepard D.S. (2013). Use of expansion factors to estimate the burden of dengue in Southeast Asia: A systematic analysis. PLoS Negl. Trop. Dis..

[6] Cardoso F.D., Rezende I.M.D., Barros E.L.T., Sacchetto L., Garcês T.C.D.C.S., Silva N.I.O. (2019). Circulation of Chikungunya virus East-Central-South Africa genotype during an outbreak in 2016–17 in Piaui State, Northeast Brazil. Rev. Inst. Med. Trop. São Paulo..

[7] Fontaine A., Diouf I., Bakkali N., Missé D., Pagès F., Fusai T., Rogier C., Almeras L. (2011). Implication of haematophagous arthropod salivary proteins in host-vector interactions. Parasites Vectors.

[8] Lee V.J., Chow A., Zheng X., Carrasco L.R., Cook A.R., Lye D.C., Ng L.C., Leo Y.S. (2012). Simple clinical and laboratory predictors of Chikungunya versus dengue infections in adults. PLoS Negl. Trop. Dis..

[9] Caminade C., McIntyre K.M., Jones A.E. (2019). Impact of recent and future climate change on vector-borne diseases. Ann. N. Y. Acad. Sci..

[10] Lee V.J., Lye D.C., Sun Y., Fernandez G., Ong A., Leo Y.S. (2008). Predictive value of simple clinical and laboratory variables for dengue hemorrhagic fever in adults. J. Clin. Virol..

[11] Paixão E.S., Teixeira M.G., Rodrigues L.C. (2018). Zika, chikungunya and dengue: The causes and threats of new and re-emerging arboviral diseases. BMJ Glob. Health.

[12] Nimmannitya S., Halstead S.B., Cohen S.N., Margiotta M.R. (1969). Dengue and chikungunya virus infection in man in Thailand, 1962–1964. Am. J. Trop. Med. Hyg..

[13] Halstead S.Á., Nimmannitya S., Cohen S. (1970). Observations related to pathogenesis of dengue hemorrhagic fever. IV. Relation of disease severity to antibody response and virus recovered. Yale J. Biol. Med..

[14] Heinze G., Schemper M. (2002). A solution to the problem of separation in logistic regression. Stat. Med..

[15] David A.M., Alexandra C.I.D., Paul R.Y. (2017). Clinical and laboratory diagnosis of dengue virus infection. J. Infect. Dis..

[16] Sanchez-Arcila J.C., Badolato-correa J., de Souza T.M.A., Paiva I.A., Barbosa L.S., Nunes P.C.G., Lima M.R.Q., dos Santos F.B., Damasco P.V., da Cunha R.V. (2020). DENV, ZIKV, and/or CHIKV-infected Brazilian patients. Intervirology.

[17] Peeling R.W., Mabe D. (2010). Point-of-care tests for diagnosing infections in the developing world. Clin. Microbiol. Inf..

[18] Reddy V., Mani R.S., Desai A., Ravi V. (2014). Correlation of plasma viral loads and presence of Chikungunya IgM antibodies with cytokine/chemokine levels during acute Chikungunya virus infection. J. Med. Virol..

[19] Chahar H.S., Bharaj P., Dar L., Guleria R., Kabra S.K., Broor S. (2009). Co-infections with chikungunya virus and dengue virus in Delhi, India. Emerg. Infect. Dis..

[20] Kumar D., Verma R.K., Singh A., Kumar M., Singh D.P., Pandey R., Krishnappa K. (2018). Evaluation of NS1, IgM ELISA and RT-PCR in diagnosis of dengue fever. Int. J. Res. Med. Sci..

[21] Gubler D., Kuno G., Sather G., Waterman S. (1985). A case of natural concurrent human infection with two dengue viruses. Am. J. Trop. Med. Hyg..

[22] Bharaj P., Chahar H.S., Pandey A., Diddi K., Dar L., Guleria R., Kabra S.K., Broor S. (2008). Concurrent infections by all four dengue virus serotypes during an outbreak of dengue in 2006 in Delhi, India. Virol. J..

[23] Myers R.M., Carey D.E. (1967). Concurrent isolation from patient of two arboviruses, Chikungunya and dengue type 2. Science.

[24] Yergolkar P.N., Tandale B.V., Arankalle V.A., Sathe P.S., Sudeep A.B., Gandhe S.S., Gokhle M.D., Jacob G.P., Hundekar S.L., Mishra A.C. (2006). Chikungunya outbreaks caused by African genotype, India. Emerg. Infect. Dis..

[25] Furuya-Kanamori L., Liang S., Milinovich G., Magalhaes R.J.S., Clements A.C., Hu W., Brasil P., Frentiu F.D., Dunning R., Yakob L. (2016). Co-distribution and co-infection of chikungunya and dengue viruses. BMC Infect. Dis..

[26] Rodriguez-Morales A.J., Villamil-Gómez W.E., Franco-Paredes C. (2016). The arboviral burden of disease caused by co-circulation and co-infection of dengue, chikungunya and Zika in the Americas. Travel Med. Infect Dis..

[27] Vogels C.B.F., Rückert C., Cavany S.M., Perkins T.A., Ebel G.D., Grubaugh N.D. (2019). Arbovirus coinfection and co-transmission: A neglected public health concern?. PLoS Biol..

[28] Rojas J.M., Avia M., Martín V., Sevilla N. (2017). IL-10: A Multifunctional cytokine in viral infections. J. Immunol. Res..

[29] Schaible H.G., von Banchet G.S., Boettger M.K., Bräuer R., Gajda M., Richter F., Hensellek S., Brenn D., Natura G. (2010). The role of proinflammatory cytokines in the generation and maintenance of joint pain. Ann. N. Y. Acad. Sci..

[30] Cronstein B.N. (2007). Interleukin-6--a key mediator of systemic and local symptoms in rheumatoid arthritis. Bull. NYU Hosp. Jt. Dis..

[31] Deshmane S.L., Kremlev S., Amini S., Sawaya B.E. (2009). Monocyte chemoattractant protein-1 [MCP-1]: An overview. J. Interferon Cytokine Res..

[32] Kelvin A.A., Banner D., Silvi G., Moro M.L., Spataro N., Gaibani P., Cavrini F., Pierro A., Rossini G., Cameron M.J. (2011). Inflammatory cytokine expression is associated with chikungunya virus resolution and symptom severity. PLoS Negl. Trop. Dis..

[33] Silva M.R., Briseño J.A.A., Upasani V., van der Ende-Metselaar H., Smit J.M., Rodenhuis-Zybert I.A. (2017). Suppression of chikungunya virus replication and differential innate responses of human peripheral blood mononuclear cells during co-infection with dengue virus. PLOS Negl. Trop. Dis..

[34] Weaver S.C., Osorio J.E., Livengood J.A., Chen R., Stinchcomb D.T. (2012). Chikungunya virus and prospects for a vaccine. Expert Rev. Vaccines.

[35] Chang A.Y., Tritsch S., Reid S.P., Martins K., Encinales L., Pacheco N., Amdur R.L., Porras-Ramirez A., Rico-Mendoza A., Li G. (2018). The cytokine profile in acute chikungunya infection is predictive of chronic arthritis 20 months post infection. Diseases.

[36] Jain J., Nayak K., Tanwar N., Gaind R., Gupta B., Shastri J.S., Bhatnagar R.K., Kaja M.K., Chandele A., Sunil S. (2017). Clinical, Serological, and virological analysis of 572 Chikungunya patients from 2010 to 2013 in India. Clin. Infect. Dis..

[37] Sathish N., Manayani D., Shankar V., Abraham M. (2002). Comparison of IgM capture ELISA with a commercial rapid immunochromatographic card test and IgM microwell ELISA for the detection of antibodies to dengue viruses. Ind. J. Med. Res..

[38] Shanthi G., Purushothaman I., Rajarajan S. (2016). Phylogenetic analysis of Dengue Virus Serotype 1 isolated from clinically suspected pediatric patients in Chennai, Tamilnadu. Int. J. Adv. Biotech. Res..

[39] Tuekprakhon A., Puiprom O., Sasaki T., Michiels J., Bartholomeeusen K., Nakayama E.E., Meno M.K., Phadungsombat J., Huits R., Ariën K.K. (2018). Broad-spectrum monoclonal antibodies against chikungunya virus structural proteins: Promising candidates for antibody-based rapid diagnostic test development. PLoS ONE.

[40] Harder T.C., Harder M., Vos H., Kulonen K., Kennedy-Stoskopf S., Liess B., Appel M.J., Osterhaus A.D. (1996). Characterization of phocid herpesvirus-1 and -2 as putative alpha- and gammaherpesviruses of North American and European pinnipeds. J. Gen. Virol..

[41] Edwards C.J., Welch S.R., Chamberlain J., Hewson R., Tolley H., Cane P.A., Lloyd G. (2007). Molecular diagnosis and analysis of Chikungunya virus. J. Clin. Virol..

[42] Amsen D., De Visser K.E., Town T. (2009). Approaches to determine expression of inflammatory cytokines. Inflamm. Cancer.

