# Supplementary material for: Comparison of Cytokine Expression Profile in Chikungunya and Dengue Co-Infected and Mono-Infected Patients’ Samples
Source: Pathogens. 2021 Feb 4;10(2):166. doi: 10.3390/pathogens10020166 (PMC7913810; doi:10.3390/pathogens10020166)
Supplement: Supplementary file 1 [file pathogens-10-00166-s001.pdf]

Supplementary data of ROC file:

AUC:

AUC	CONTROL	P value	CHIKV	P value	DENV	P value	CHIK & DENV	P value
IL-2	0.9276	<<0.0001	0.9025	<0.0001	0.9111	<0.0001	1.0000	<0.0001
IL-8	0.9263	<<0.0001	0.8701	<0.0002	0.9013	<0.0001	0.9227	<0.0001
IL-10	0.9885	<<0.0001	0.9885	<0.0001	0.9852	<0.0001	0.9951	<0.0001
IL-12	0.7188	<0.0277	0.9737	<0.0001	0.9079	<0.0001	0.9539	<0.0001
IFN-α	0.9474	<0.0001	0.8274	<0.0007	0.6801	<0.0776	0.6000	<0.2678

SENSTIVITY:

Senstivity %	CONTROL	CHIKV	DENV	CHIK & DENV
IL-2	75.82	99.10	69.27	69.51
IL-8	71.99	69.98	70.67	73.03
IL-10	71.06	75.30	74.315	75.30
IL-12	52.53	76.91	68.84	75.89
IFN-α	73.684	64.55	59.007	54.877

Specificity:

SPECIFICITY %	CONTROL	CHIKV	DENV	CHIK & DENV
IL-2	66.66	38.09	73.12	78.90
IL-8	73.85	69.21	74..10	71.8
IL-10	78.57	75.12	72.25	75.72
IL-12	53.68	74.19	76.25	72.9
IFN-α	70.60	69.09	61.49	55.36
